# Trop2 Expression in Extramammary Paget’s Disease and Normal Skin

**DOI:** 10.3390/ijms22147706

**Published:** 2021-07-19

**Authors:** Takamichi Ito, Keiko Tanegashima, Yuka Tanaka, Hiroki Hashimoto, Maho Murata, Yoshinao Oda, Yumiko Kaku-Ito

**Affiliations:** 1 Department of Dermatology, Graduate School of Medical Sciences, Kyushu University, Fukuoka 812-8582, Japan; mpsyp388@yahoo.co.jp (K.T.); yukat53@med.kyushu-u.ac.jp (Y.T.); h-hashi@dermatol.med.kyushu-u.ac.jp (H.H.); muratama@dermatol.med.kyushu-u.ac.jp (M.M.); kyumiko@dermatol.med.kyushu-u.ac.jp (Y.K.-I.); 2 Department of Anatomic Pathology, Graduate School of Medical Sciences, Kyushu University, Fukuoka 812-8582, Japan; oda@surgpath.med.kyushu-u.ac.jp

**Keywords:** trophoblast cell surface antigen 2 (Trop2), extramammary Paget’s disease, sacituzumab govitecan, targeted therapy

## Abstract

Extramammary Paget’s disease (EMPD) is a rare skin cancer arising in the apocrine gland-rich areas. Most EMPD tumors are dormant, but metastatic lesions are associated with poor outcomes owing to the lack of effective systemic therapies. Trophoblast cell surface antigen 2 (Trop2), a surface glycoprotein, has drawn attention as a potential therapeutic target for solid tumors. Sacituzumab govitecan, an antibody–drug conjugate of Trop2, has recently entered clinical use for the treatment of various solid cancers. However, little is known about the role of Trop2 in EMPD. In this study, we immunohistochemically examined Trop2 expression in 116 EMPD tissue samples and 10 normal skin tissues. In normal skin, Trop2 was expressed in the epidermal keratinocytes, inner root sheaths, and infundibulum/isthmus epithelium of hair follicles, eccrine/apocrine glands, and sebaceous glands. Most EMPD tissues exhibited homogeneous and strong Trop2 expression, and high Trop2 expression was significantly associated with worse disease-free survival (*p* = 0.0343). These results suggest the potential use of Trop2-targeted therapy for EMPD and improve our understanding of the skin-related adverse effects of current Trop2-targeted therapies such as sacituzumab govitecan.

## 1. Introduction

Extramammary Paget’s disease (EMPD) is a rare skin cancer that mainly affects apocrine sweat gland-rich areas in elderly people [1,2]. The disease most frequently arises in the anogenital area and less commonly in the axillary area [2,3,4]. EMPD typically affects Caucasian woman and Asian men older than 60 years [3,4,5,6]. Primary EMPD arises as an intraepithelial neoplasm of the epidermis and can be distinguished from mammary Paget’s disease (a type of breast cancer) and secondary EMPD (direct invasion from the visceral cancers including colorectal, vaginal, and urothelial cancers) clinically and immunohistochemically [1,4]. Most patients with EMPD have a good prognosis owing to the slow-growing nature of the disease given that tumors arise in the epidermis and remain dormant as in situ lesions for prolonged periods [1,7,8]. However, EMPD lesions show infiltrative erythema with crust and scale and clinically mimick many other benign inflammatory skin diseases including contact dermatitis, eczema, and superficial fungal infections, which leads to misdiagnosis or diagnostic delay [4]. Approximately 15–40% of EMPD lesions exhibit dermal invasion in their clinical course, increasing the risk of lymph node and distant metastasis [2,4]. Although complete surgical removal is the treatment of choice for resectable EMPD, this is sometimes difficult to achieve because of the inconspicuous tumor border and anatomical constraints [9,10]. The prognosis of unresectable EMPD is poor once the tumor metastasizes because of the lack of effective systemic therapies [4,11,12,13,14]. Although molecular targeted therapy (e.g., Her-2 inhibitors) with or without conventional chemotherapy has been applied, the efficacy of these treatments is unsatisfactory. A novel therapeutic option is desirable [4,11].

Trophoblast cell surface antigen 2 (Trop2), also known as tumor-associated calcium signal transducer (Tacstd2), is a surface glycoprotein originally identified in human placental trophoblasts [15,16,17]. Trop2 is highly expressed in various cancer cells, such as pancreatic [18], gastric [19], lung [15], and colorectal cancer cells [20,21,22], and it regulates cancer proliferation, migration, invasion, and metastasis [23,24,25,26,27,28]. Because Trop2 overexpression is associated with poor survival in patients with solid tumors, Trop2 has been considered a potential target for anticancer therapy [21,29]. In studies of breast and lung cancers, Trop2 inhibition exerted anticancer effects [30,31]. Recently, an antibody–drug conjugate (ADC) targeting Trop2 was used in clinical trials in lung, urothelial, breast, and other miscellaneous epithelial cancers, and clinical benefits were observed [32,33,34,35,36]. However, little is known about the role of Trop2 in EMPD. In the current study, we analyzed the expression of Trop2 in primary EMPD using clinical samples from 116 patients and examined the correlation between Trop2 overexpression and patient prognosis, thereby clarifying whether Trop2 could be a therapeutic target for EMPD. We also examined the localization of Trop2 in normal skin and appendages to better understand the skin adverse effects of Trop2-targeting ADCs.

## 2. Results

### 2.1. Patients

The comprehensive demographic data of 116 patients with primary EMPD are presented in Table 1. The mean patient age was 73.2 years (range, 42–91). The study cohort included 71 men (61.2%) and 45 women (38.8%). The most common primary tumor site was the anogenital area (94.8%), followed by the axilla (5.2%). The TNM stage was defined in accordance with the EMPD-specific staging system proposed by Ohara et al. in 2016 [7]. The majority of the patients (85.3%) presented with TNM stage I or II lesions (no lymph node or distant metastasis), whereas 14.7% of patients had stage III or IV disease (presence of at least one lymph node or distant metastatic lesion). The tumor thickness (TT) in the patient cohort was as follows: TT ≤ 1 mm in 72.4% of patients; 1 mm < TT ≤ 2 mm in 12.1% of patients; 2 mm < TT ≤ 4 mm in 5.2% of patients; and TT > 4 mm in 9.5% of patients.

### 2.2. Trop2 Expression in Normal Skin and Skin Appendages

**Figure 1:**
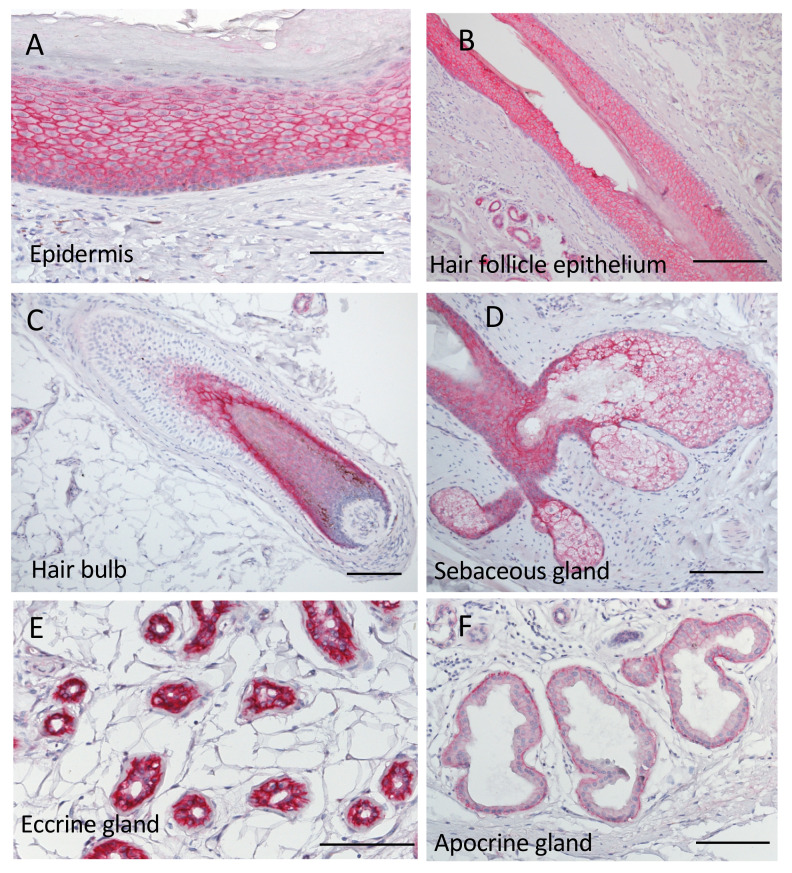
Representative histopathological images of Trop2 staining in human normal skin and skin appendages. Positive signals are presented in red. (**A**) Epidermis. The epidermal keratinocytes exhibited negative to faint staining in the basal layer, strong membranous and weak cytoplasmic staining in the spinous layer, and no membranous or cytoplasmic Trop2 expression in the granular layer. A few cells of granular layer had weak nuclear staining. (**B**) Hair follicle epithelium. Keratinocytes in the infundibulum and isthmus displayed a similar expression pattern as that observed in the epidermis. (**C**) Hair bulb. Trop2 staining was positive in the inner root sheath (membranous 3+, cytoplasmic 3+), matrix (membranous 1+, cytoplasmic 1+), and cortex (membranous 1+, cytoplasmic 1+) but negative in hair germ cells and the outer root sheath. (**D**) Sebaceous gland. Trop2 staining was strongly positive (membranous 3+, cytoplasmic 3+). (**E**) Eccrine gland. Trop2 staining was strongly positive (membranous 3+, cytoplasmic 3+). (**F**) Apocrine gland. Trop2 staining was moderately positive (membranous 2+, cytoplasmic 1+). Bars indicate 100 μm. Trop2, trophoblast cell surface antigen 2.

We first examined Trop2 expression and localization in normal human skin and skin appendages. Figure 1 presents representative histopathological images of Trop2. In normal skin and appendages, Trop2 expression was observed in a membranous, cytoplasmic, and/or nuclear pattern (Table 2). Epidermal keratinocytes displayed negative to faint staining in the basal layer, strong membranous and weak cytoplasmic staining in the spinous layer, and no membranous or cytoplasmic Trop2 expression in the granular layer. A few cells of the granular layer exhibited weak nuclear staining (Figure 1A,B). In hair follicles, Trop2 displayed a site-specific distribution (Figure 1C). Trop2 staining was positive in the infundibular epithelium (membranous 3+, cytoplasmic 1+), inner root sheath (membranous 3+, cytoplasmic 3+), matrix (membranous 1+, cytoplasmic 1+), and cortex (membranous 1+, cytoplasmic 1+) but negative in hair germ cells and the outer root sheath. In the other skin appendages, Trop2 was strongly positive in sebaceous (membranous 3+, cytoplasmic 3+; Figure 1D) and eccrine glands (membranous 3+, cytoplasmic 3+; Figure 1E), and moderately in apocrine glands (membranous 2+, cytoplasmic +; Figure 1F).

### 2.3. Trop2 Expression in Extramammary Paget’S Disease

**Figure 2:**
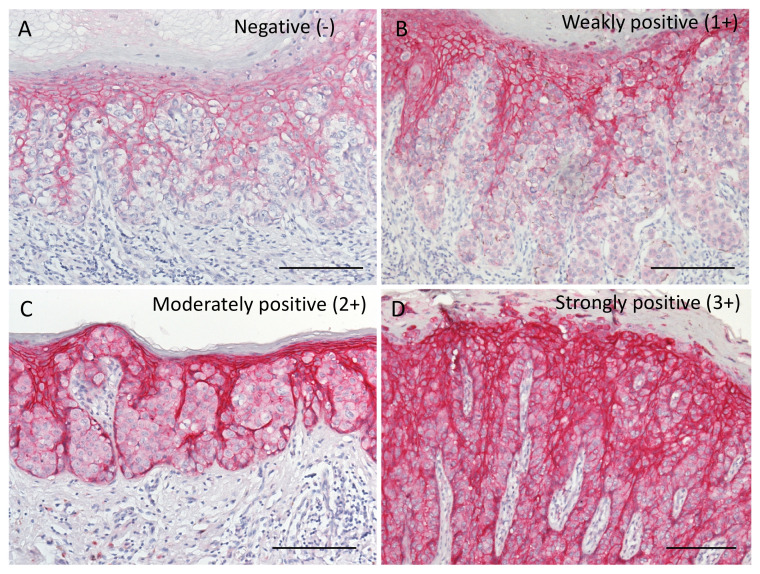
(**A**–**D**) Representative histopathological images of (**A**) negative (−), (**B**) weakly positive (1+), (**C**) moderately positive (2+), and (**D**) strongly positive (3+) staining for Trop2. Positive Trop2 signals were mainly found in the cytoplasm and on the membranes of tumor cells. Bars indicate 100 μm. Trop2, trophoblast cell surface antigen 2.

We next examined Trop2 expression in EMPD tissue samples. Representative images of negative, weakly positive (1+), moderately positive (2+), and strongly positive (3+) Trop2 staining are presented in Figure 2. Positive Trop2 signals were mainly found in the cytoplasm and on the membranes of tumor cells (Figure 2). We divided the samples into two groups based on the median H-score: Trop2-low (H-score < 100) and Trop2-high (H-score ≥ 100).

**Figure 3:**
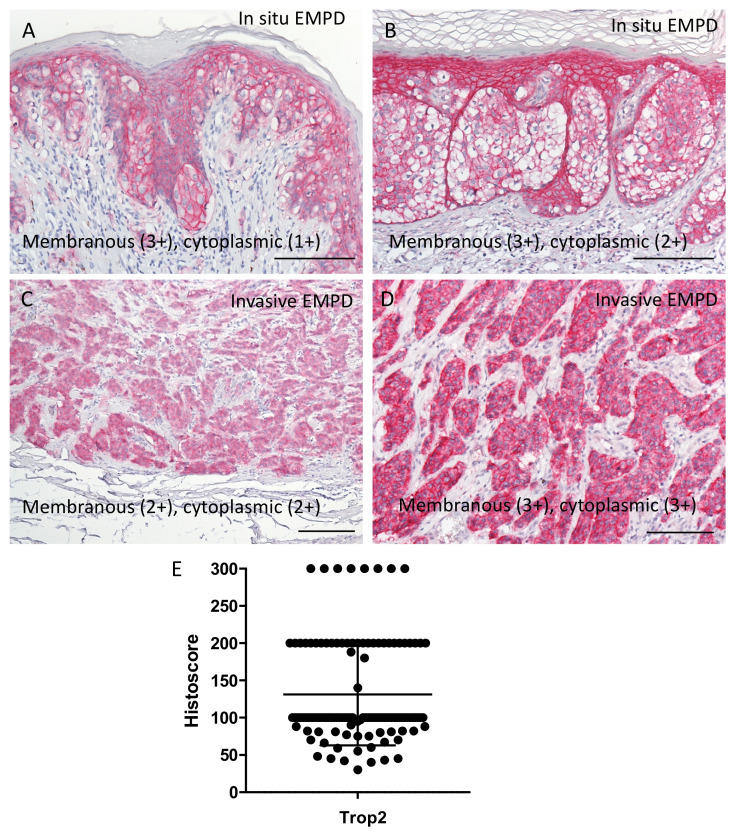
(**A**–**D**) Other histopathological images of Trop2 staining. In situ (**A**,**B**) and invasive (**C**,**D**) EMPD. Staining patters were variable. Cytoplasmic staining was observed in most EMPD tissues with or without membranous staining. Cytoplasmic staining was obscure in some mucin-rich cells (**B**). (**E**) Histoscores of Trop2 staining. All EMPD tissues had positive Trop2 staining in at least some areas. Bars indicate 100 μm. Trop2, trophoblast cell surface antigen 2; EMPD, extramammary Paget’s disease.

Representative images of Trop2 in in-situ and invasive EMPD are presented in Figure 3. Of note, all EMPD tissues had positive Trop2 staining in some regions (Figure 3A–E). The staining patters were variable. Cytoplasmic staining was observed in most EMPD lesions with or without membranous staining, whereas cytoplasmic staining was obscure in some mucin-rich cells (Figure 3B).

### 2.4. Association between Trop2 and Clinicopathological Factors in EMPD

The associations between immunohistochemical Trop2 expression and clinicopathological factors were examined, as presented in Table 3. TT was categorized as ≤1 mm or >1 mm in accordance with previous reports [4,30]. In total, 86 (74.1%) and 30 samples (25.9%) were categorized into the Trop2-high and Trop2-low groups, respectively. No significant differences in background data (age, sex, tumor site, TNM stage, and TT) were observed between the two groups (Table 3).

### 2.5. Prognostic Impact of Trop2 Expression

**Figure 4:**
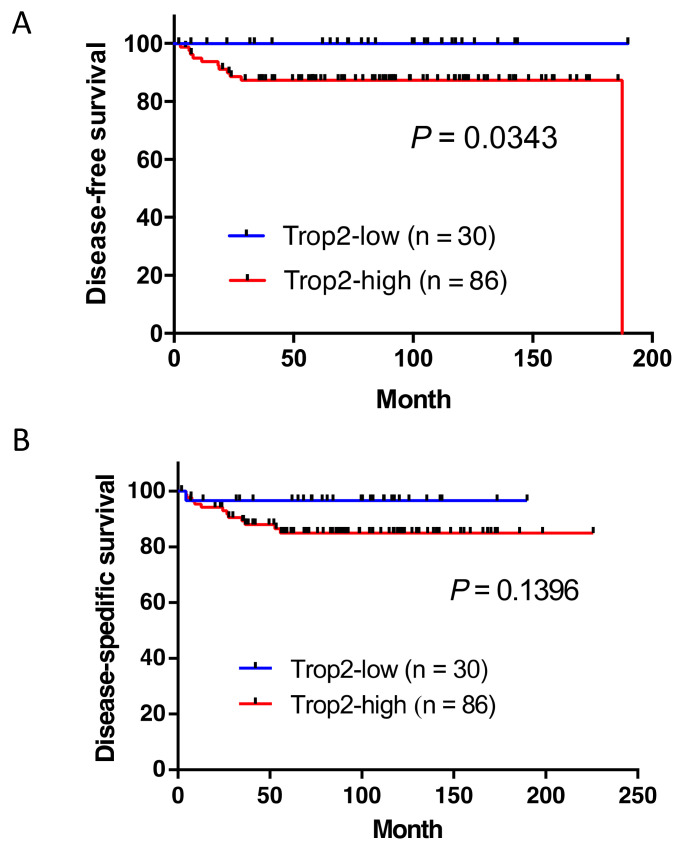
Kaplan–Meier survival curves of patients with Trop2-low and Trop2-high extramammary Paget disease (EMPD). (**A**) Patients with high-Trop2 EMPD had significantly shorter disease-free survival than those with low-Trop2 (*p* = 0.0343). (**B**) For disease-specific survival, the difference between the two group did not reach statistical significance (*p* = 0.1396). Trop2, trophoblast cell surface antigen 2.

**Figure 5:**
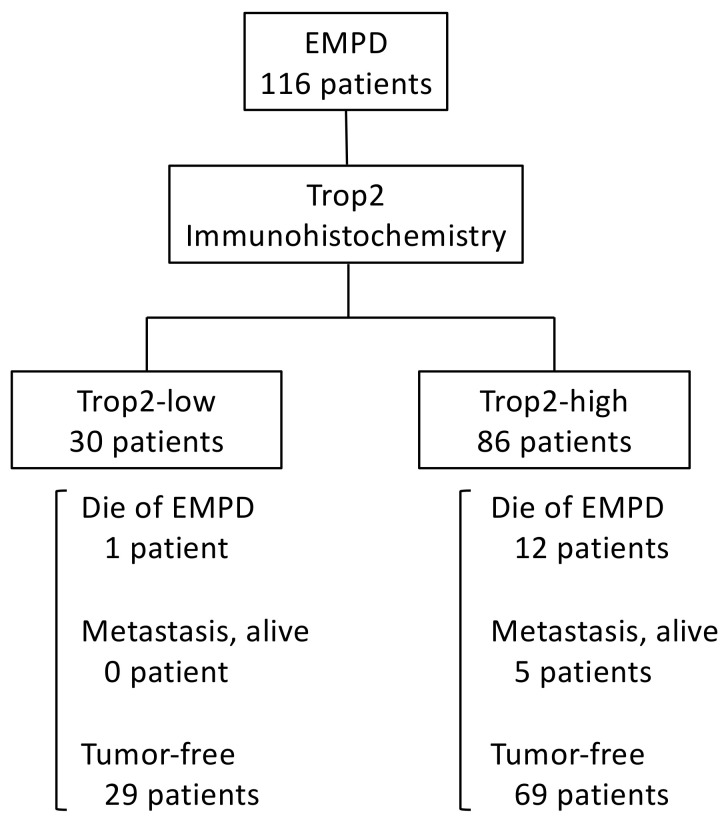
A schematic diagram of patients. EMPD, extramammary Paget’s disease; Trop2, trophoblast cell surface antigen 2.

We then compared survival between the Trop2-high and Trop2-low groups. The Kaplan–Meier survival curves are presented in Figure 4. Interestingly, patients with high-Trop2 EMPD had significantly shorter disease-free survival (DFS) than those with low-Trop2 EMPD (*p* = 0.0343), whereas the difference in disease-specific survival (DSS) between the groups did not reach the statistical significance (*p* = 0.1396). A schematic diagram of the patients is presented in Figure 5.

## 3. Discussion

Trop2, encoded by the Tacstd2 gene, is a surface glycoprotein originally identified in human placental trophoblasts [16,17]. It is involved in a variety of cell signaling pathways, including proliferation, survival, self-renewal, and invasion [37]. Trop2 contains a hydrophobic transmembrane domain (extracellular domain) and an intracellular domain, and it is cleaved into the two parts via regulated intramembrane proteolysis [38]. Following cleavage, the intracellular domain is released into the nucleus. Conversely, the extracellular domain is released into the cytoplasm, or it lingers on the membrane. In the nucleus, β-catenin colocalizes with the Trop2 intracellular domain, which upregulates cyclin D1 and c-Myc [23,38]. Trop2 is highly expressed in a variety of cancers, and its high expression influences metastasis by regulating epithelial-to-mesenchymal transition and leads to a dismal prognosis [30,31,39,40]. Consequently, Trop2 has attracted attention as a potential target for anticancer therapy. Blockade of Trop2 using anti-Trop2 antibodies resulted in anticancer activity in head and neck squamous cell carcinoma [41] and pancreatic cancer [42], and suppression of Trop2 by the natural product curcumin inhibited cell proliferation and motility in bladder cancer cells [43]. However, Trop2 expression in EMPD and normal skin has not been examined. In this study, we observed specific Trop2 expression in keratinocytes, particularly in the spinous layer. Interestingly, strong Trop2 expression was observed in the inner root sheaths of the hair follicles, sweat gland epithelium, and sebaceous glands. These findings suggest the potential efficacy of recently developed Trop2-targeted therapies for malignant tumors derived from skin epidermis and skin appendages. This detailed localization of Trop2 should improve the understanding of the adverse effects of target therapies on the skin.

Interestingly, all EMPD tissues examined in this study expressed Trop2 in at least some areas, with most lesions (71.6%) having a proportion score of 100%. Furthermore, Trop2-high EMPD was linked to shorter DFS. These findings support Trop2 inhibition as a novel treatment strategy for EMPD. In fact, the depletion of Trop2 inhibited tumor proliferation in breast cancer, which shares some tumor characteristics with EMPD [4]. In clinical settings, EMPD is curable when surgically eradicated in the early stage because the tumors generally exhibit slow growth and a long dormant phase [4,44]. However, complete surgical removal is sometimes difficult because of diagnostic delays or anatomical constraints, leading to an increased risk of metastasis [4,10]. Conventional chemotherapy with taxanes, platinum-containing drugs, 5-fluorouracil, epirubicin, vincristine, and mitomycin C has been to treat metastatic EMPD, but the efficacy is unsatisfactory [4,11]. Targeted therapy (e.g., trastuzumab [anti-Her2 antibody]) has opened a new pathway of treatment, but a novel anticancer strategy for EMPD is still required.

As a Trop2-targeted therapy, sacituzumab govitecan has recently entered clinical use [35,36]. Sacituzumab govitecan is an ADC consisting of a fully humanized IgG1 anti-Trop2 antibody and the active metabolite of irinotecan (SN-38), a topoisomerase I inhibitor. The antibody is linked to SN-38 by a hydrolysable linker, which causes the release of drug molecules into the tumor microenvironment, thereby killing adjacent tumor cells (bystander effect) [45,46]. Sacituzumab govitecan was first approved by the US Food and Drug Administration for the treatment of triple-negative breast cancer and later authorized for treating lung cancer and urothelial carcinoma [45]. Bardia et al. recently reported the results of a phase III trial for relapsed or refractory metastatic triple-negative breast cancer (NCT02574455) [35]. In the randomized trial, both progression-free survival and overall survival were significantly longer with the addition of sacituzumab govitecan than with single-agent chemotherapy [35]. In a phase I/II basket trial (NCT01631552), sacituzumab govitecan also demonstrated antitumor efficacy and acceptable tolerability in patients with miscellaneous advanced epithelial cancers (e.g., small-cell lung cancer, colorectal cancer, esophageal cancer, endometrial cancer, pancreatic ductal adenocarcinoma, prostate cancer) [36]. In the trial, patients were enrolled regardless of their Trop2 expression levels, and Trop2 was validated as a broad target in solid tumors. Our immunohistochemical results revealed strong Trop2 expression in EMPD, suggesting EMPD is a good candidate for sacituzumab govitecan. Furthermore, strong Trop2 expression in hair follicles accords well with the fact that alopecia was a common (occurred in 40.4% of patients) treatment-related adverse event in the basket trial [36].

Besides the potential biases inherent in the retrospective design, one limitation of this study is that we did not address the molecular mechanisms of Trop2, and the roles of Trop2 in EMPD are still unknown because no cell line of EMPD has been established.

In summary, we demonstrated the detailed expression of Trop2 in normal skin and skin appendages. Most EMPD tumors exhibited high Trop2 expression, which was significantly correlated with worse DFS. Trop2-targeted therapies, such as sacituzumab govitecan, could be new treatment options for unresectable EMPD.

## 4. Materials and Methods

### 4.1. Ethics Statement

We conducted this retrospective study in accordance with the concepts enshrined in the Declaration of Helsinki. This study was approved by the Kyushu University Institutional Ethics Committee (30-363; 27 November 2018). Written informed consent was received from the patients prior to their inclusion in the study.

### 4.2. Patients

We retrieved data for 116 patients with primary EMPD lesions who were treated at the Department of Dermatology, Kyushu University (Fukuoka, Japan) between January 1997 and December 2018. At least three experienced dermatopathologists confirmed the diagnosis. Secondary EMPD, which involved direct invasion from the visceral organs, was carefully excluded. The clinical and demographic data of all patients were collected from patients’ files and analyzed.

### 4.3. Immunohistochemistry

We examined 116 EMPD tissue samples and 10 normal skin samples. All formalin-fixed (24 h in 10% buffered formalin), paraffin-embedded tissues were obtained from the archives of our hospital. Immunohistochemical staining was performed as reported previously [47,48]. Briefly, tissue samples were cut into 4-μm sections. For Trop2, antigen was retrieved using Heat Processor Solution pH 9 (Nichirei Biosciences, Tokyo, Japan) at 100 °C for 40 min. The primary antibody was diluted with Dako REAL Antibody Diluent (s2022; Dako Denmark A/S, Glostrup, Denmark). The sections were incubated with rabbit anti-human Trop2 (1:1000, ab214488; Abcam, Cambridge, UK) as the primary antibody for 30 min at room temperature followed by incubation with N-Histofine Simple Stain AP MULTI (414261; Nichirei Biosciences) as the secondary antibody for 90 min at room temperature. Immunoreactions were detected using FastRed II (415261; Nichirei Biosciences) as a chromogen, and specimens were counterstained using hematoxylin.

### 4.4. Evaluation of Trop2 Immunohistochemical Staining

The immunohistochemical results were evaluated by a semiquantitative approach using a histochemical scoring system (H-score) [49]. The intensity of staining was graded as follows: no staining (0), weakly positive (1+), moderately positive (2+), and strongly positive (3+). The epidermis was used as an internal control, and its score was 3+. The H-score of Trop2 was calculated as the percentage of positive cells (0–100%, either cytoplasmic or membranous staining) multiplied by the staining intensity (0–3+), with the final score ranging from 0 to 300. For samples with both membranous and cytoplasmic staining, we recorded the stronger intensity of staining. Two independent dermatologists (T.I. and Y.K-I.) who were blinded to the patients’ clinical information assessed the sections. Images were taken using an ECLIPSE 80i microscope (Nikon, Tokyo, Japan).

### 4.5. Statistical Analysis

All statistical analyses were performed using GraphPad Prism version 8.3 (GraphPad Software, San Diego, CA, USA) and JMP Pro version 16.0.0 (SAS Institute, Cary, NC, USA). To analyze the relationship between two categorical variables, Fisher’s exact test was used. DSS and DFS were calculated using the Kaplan–Meier method and the log-rank test. For multivariate survival analysis, we used the multivariate Cox proportional hazards regression model. *p* < 0.05 indicated a statistically significant difference.

## Figures and Tables

**Table 1:** Demographic data of 116 patients with primary extramammary Paget’s disease.

Parameters	
**Age**, **years**	
Mean	73.2
Median	73
Range	42–91
**Sex**	
Male	71 (61.2%)
Female	45 (38.8%)
**Tumor site**	
Anogenital area	110 (94.8%)
Axilla	6 (5.2%)
**TNM stage**	
I–II	99 (85.3%)
III–IV	17 (14.7%)
**Tumor thickness** (**TT**)	
TT ≤ 1 mm	84 (72.4%)
1 mm < TT ≤ 2 mm	14 (12.1%)
2 mm < TT ≤ 4 mm	6 (5.2%)
TT > 4 mm	11 (9.5%)
Unknown	1 (0.9%)

**Table 2:** Trop2 expression in normal skin and appendages.

Epidermis	
Granular cells	n(1+)
Spinous cells	m(3+), c(1+)
Basal cells	(-)
**Hair**	
Hair germ cells	(-)
Matrix	m(1+), c(1+)
Cortex	m(1+), c(1+)
Inner root sheath	m(3+), c(3+)
Outer root sheath	(-)
Infundibulum/isthmus	m(3+), c(1+)
**Eccrine gland**/**duct**	m (3+), c(3+)
**Apocrine gland**/**duct**	m(2+), c(1+)
**Sebaceous gland**/**duct**	m(3+), c(3+)

Trop2, trophoblast cell surface antigen 2; c, cytoplasmic; m, membranous.

**Table 3:** Clinicopathological factors associated with Trop2 expression.

	Trop2 Expression		
Parameters	Low	High	*p*-Value
**Age** (**years**)			
	71.6 ± 9.54	73.7 ± 9.23	0.33
**Sex**			
Male	19	52	0.83
Female	11	34	
**Tumor site**			
Anogenital area	29	81	1.00
Axilla	1	5	
**TNM stage**			
I–II	28	71	0.23
III–IV	2	15	
Tumor thickness *			
≤1 mm	25	59	0.16
>1 mm	5	26	
Total	30 (25.9%)	86 (74.1%)	

* excluding one patient with unknown tumor thickness; Trop2, trophoblast cell surface antigen 2.

## Data Availability

The data presented in this study are available in the main text.
